# Supplementary material for: Quantitative Analysis and Stability Study on Iridoid Glycosides from Seed Meal of Eucommia ulmoides Oliver
Source: Molecules. 2022 Sep 12;27(18):5924. doi: 10.3390/molecules27185924 (PMC9501183; doi:10.3390/molecules27185924)
Supplement: Supplementary file 1 [file molecules-27-05924-s001.zip › molecules-1892192-supplementary.pdf]

Supplementary Materials to:

Quantitative Analysis and Stability Study on Iridoid Glycosides from seed meal of *Eucommia ulmoides* Oliver

Lulu Ma [1†], Ning Meng [1†], Benyu Liu [1], Changjian Wang [1], Xin Chai [1,2], Shan Huang [3*], Huijuan Yu [1,2*], and Yuefei Wang [1,2*]

[1] State Key Laboratory of Component-based Chinese Medicine, Tianjin Key Laboratory of TCM Chemistry and Analysis, Tianjin University of Traditional Chinese Medicine, Tianjin 301617, China
[2] Haihe Laboratory of Modern Chinese Medicine, Tianjin 301617, China
[3] Department of Pharmacy, Qingdao University of Science & Technology, Qingdao 266000, China
* Correspondence: huangshan@qust.edu.cn (S.H.); huijuanyu@tjutcm.edu.cn (H.Y.); wangyf0622@tjutcm.edu.cn (Y.W.)
Tel.: +86-22-59596366 (H.S., H.Y. & Y.W.)
† These authors contributed equally to this work.

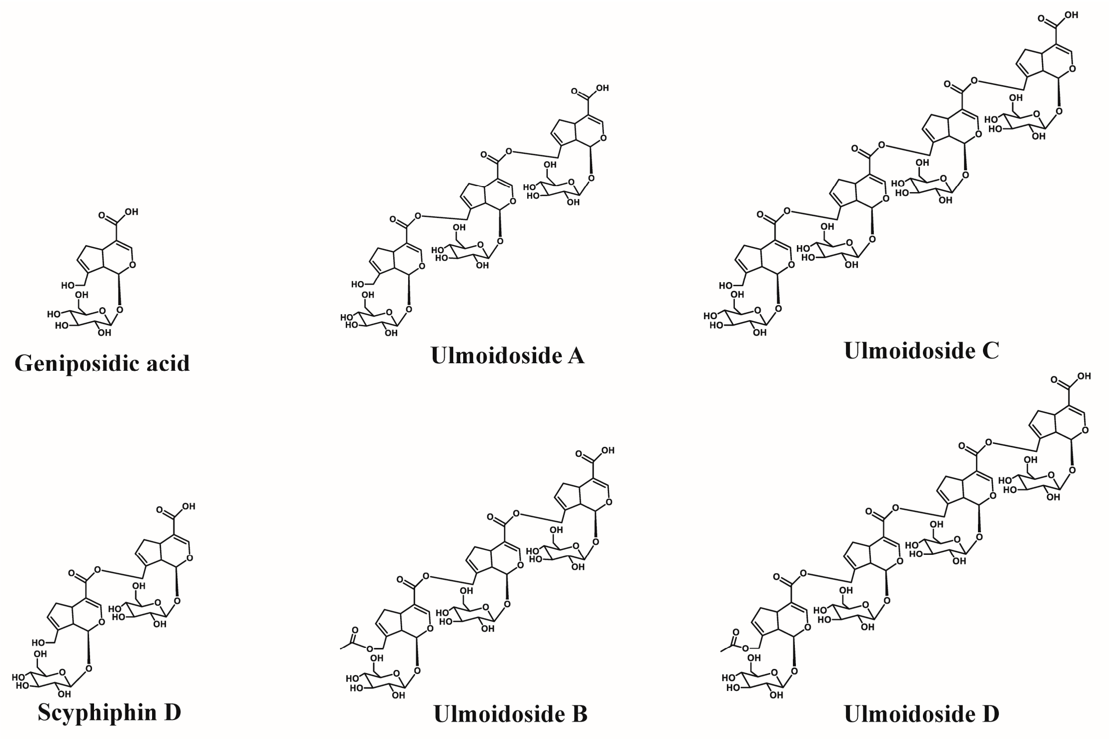

Figure S1. Structures of six natural iridoid compounds isolated from seed meal of *E. ulmoide* Oliv..

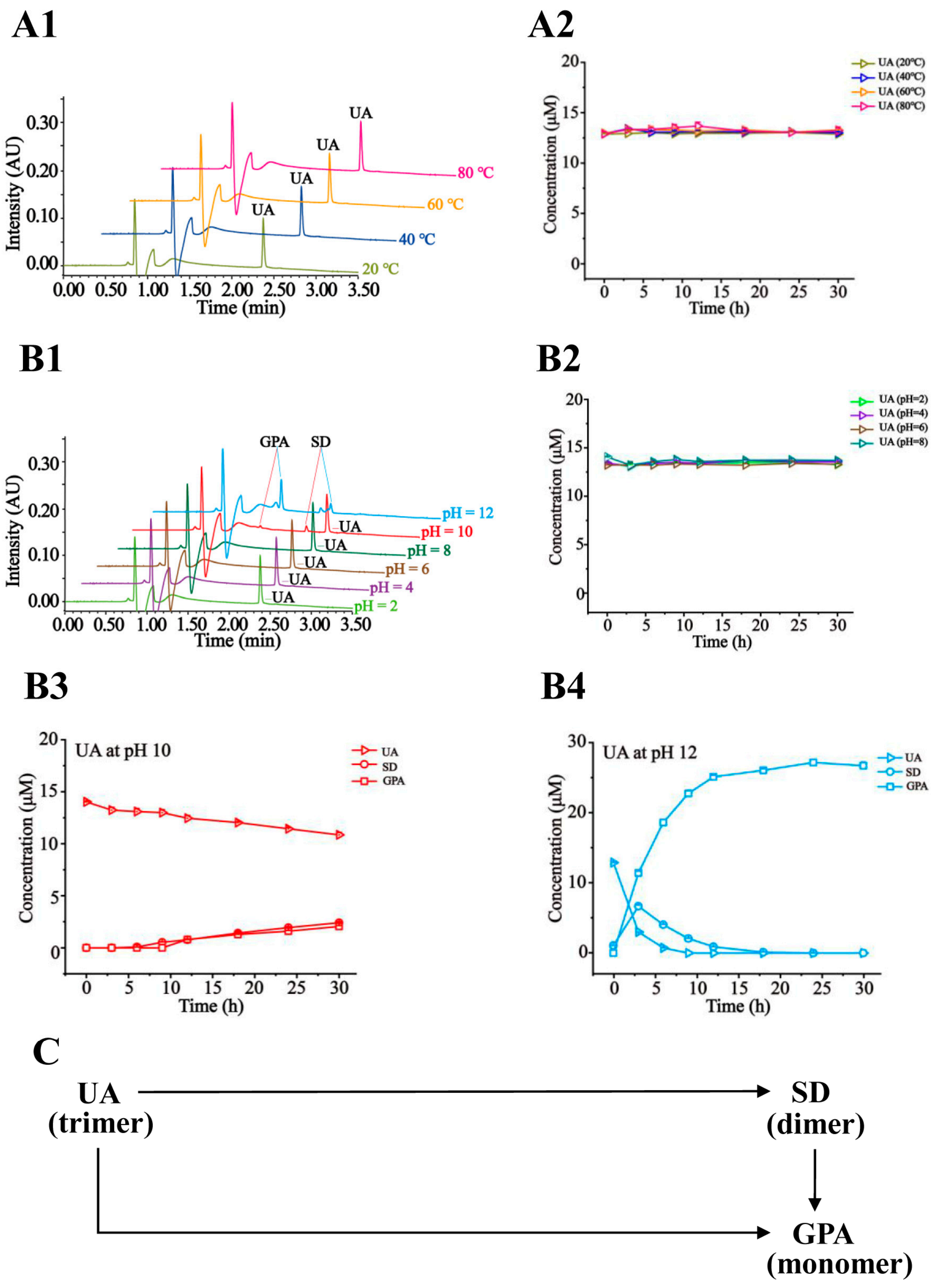

Figure S2. Representative UPLC chromatograms of UA in the tested solution exposed to different temperatures (**A1**) and pH levels (**B1**) after 24 h; Time-concentration curves of UA at different temperatures (**A2**) and pH 2 ~ 8 (**B2**); Time-concentration curves of UA and the degraded products at pH 10 (**B3**) and pH 12 (**B4**); The proposed degradation pathways of UA (**C**).

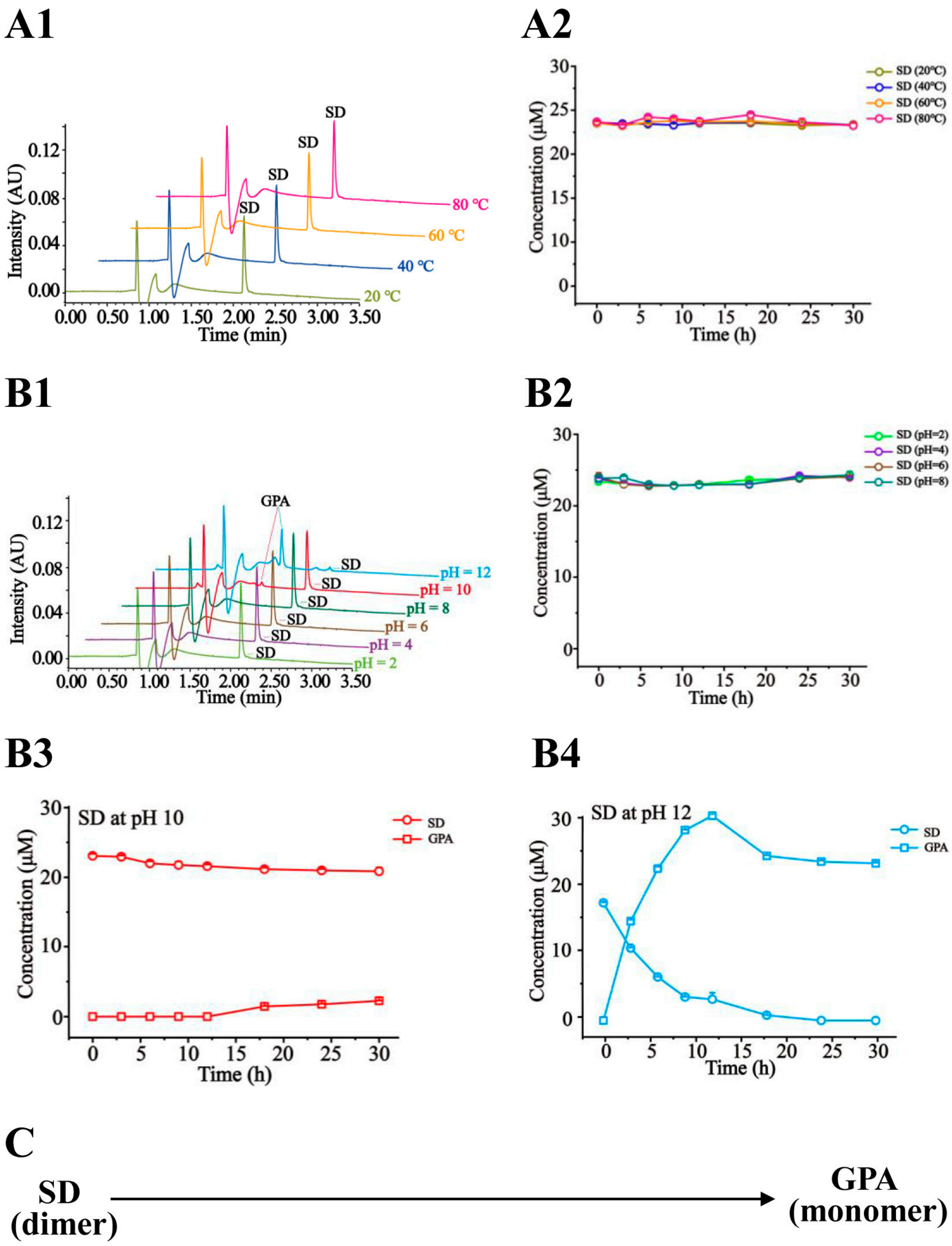

Figure S3. Representative UPLC chromatograms of SD in the tested solution exposed to different temperatures (**A1**) and pH levels (**B1**) after 24 h; Time-concentration curves of SD at different temperatures (**A2**) and pH 2 ~ 8 (**B2**); Time-concentration curves of SD and the degraded products at pH 10 (**B3**) and pH 12 (**B4**); The proposed degradation pathways of SD (**C**).

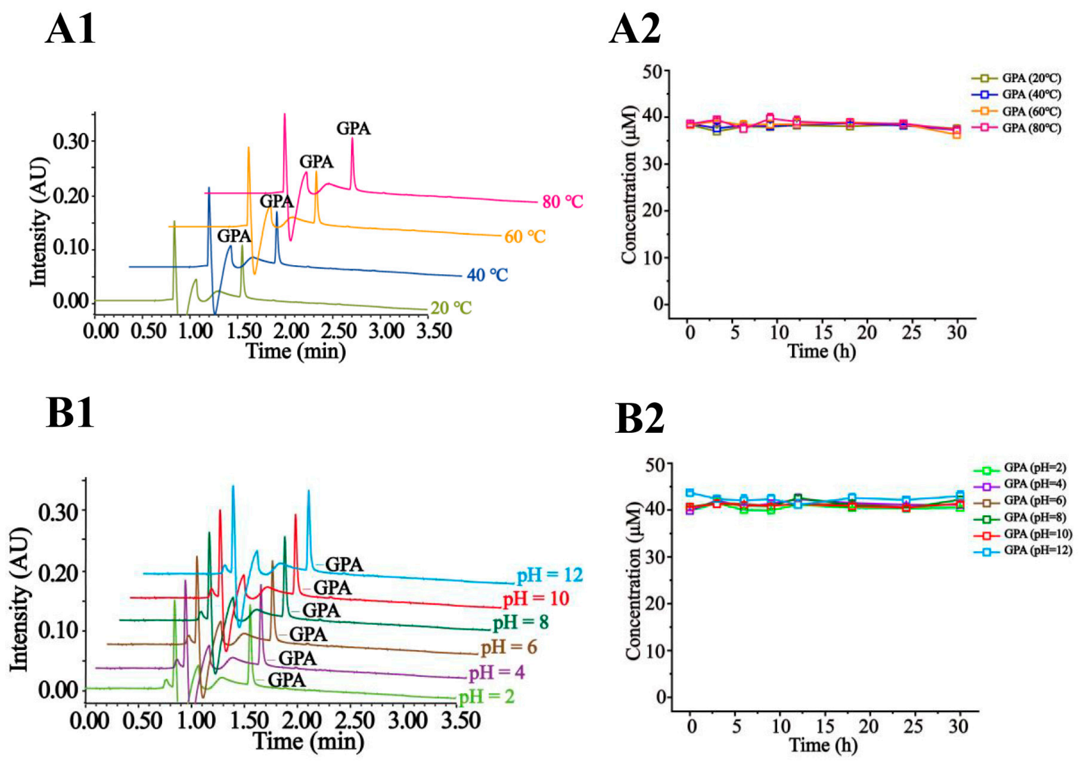

Figure S4. Representative UPLC chromatograms of GPA in the tested solution exposed to different temperatures (**A1**) and pH levels (**B1**) after 24 h; Time-concentration curves of GPA at different temperatures (**A2**) and pH 2 ~ 12 (**B2**).

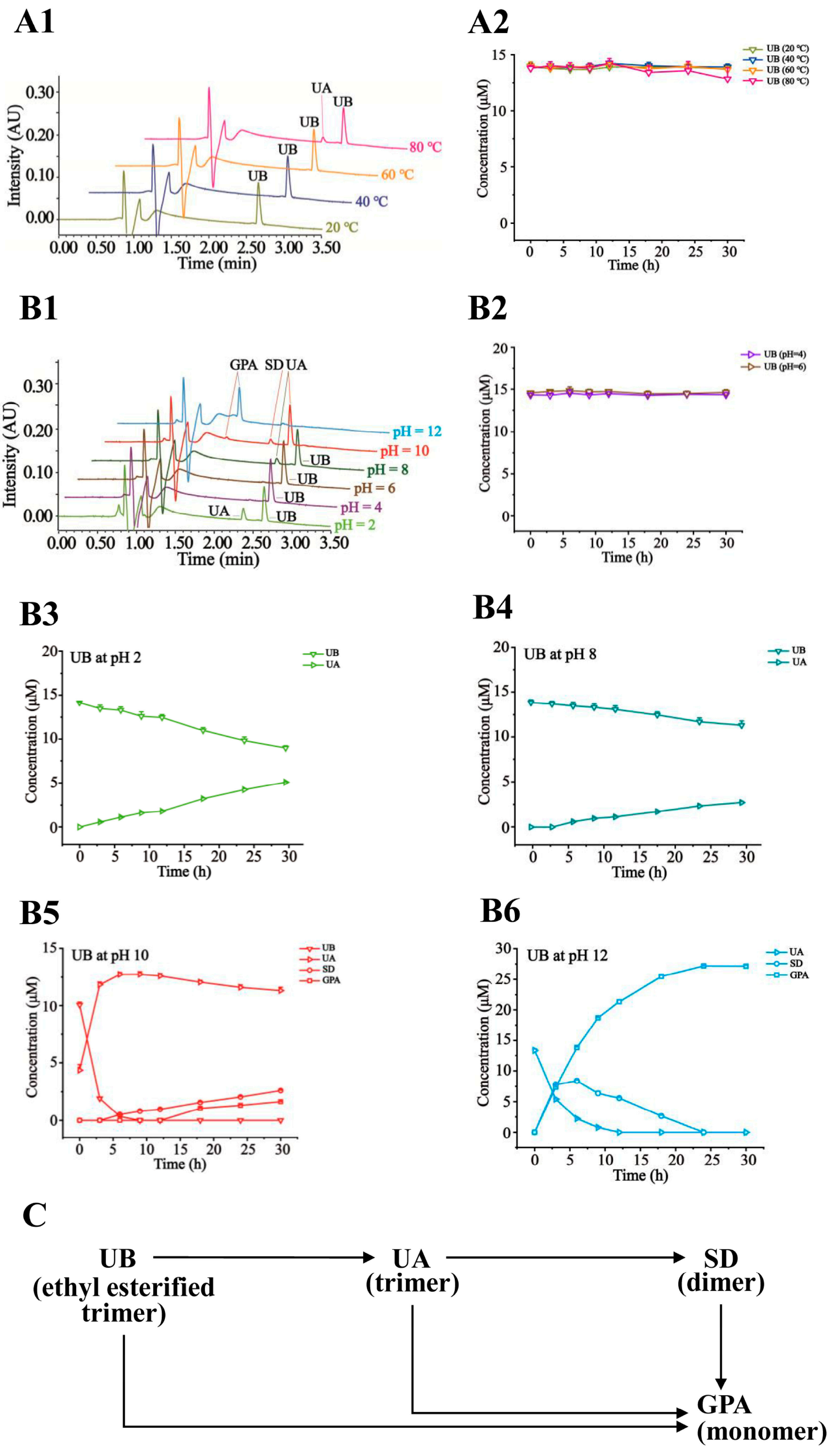
A1
Intensity (AU)
Time (min)
UA
UB
80 °C
60 °C
40 °C
20 °C
A2
UB (20 °C)
UB (40 °C)
UB (60 °C)
UB (80 °C)
Concentration (μM)
Time (h)
B1
GPA
SD
UA
UB
pH = 12
pH = 10
pH = 8
pH = 6
pH = 4
pH = 2
Intensity (AU)
Time (min)
B2
UB (pH=4)
UB (pH=6)
Concentration (μM)
Time (h)
B3
UB at pH 2
UB
UA
Concentration (μM)
Time (h)
B4
UB at pH 8
UB
UA
Concentration (μM)
Time (h)
B5
UB at pH 10
UB
UA
SD
GPA
Concentration (μM)
Time (h)
B6
UB at pH 12
UA
SD
GPA
Concentration (μM)
Time (h)
C
UB
(ethyl esterified
trimer)
UA
(trimer)
SD
(dimer)
GPA
(monomer)

Figure S5. Representative UPLC chromatograms of UB in the exposed solution at different temperatures (**A1**) and pH levels (**B1**) for 24 h; Time-concentration curves of UB at different temperatures (**A2**) and pH 4 ~ 6 (**B2**); Time-concentration curves of UB and the degraded products at pH 2 (**B3**), pH 8 (**B4**), pH 10 (**B5**), and pH 12 (**B6**); The proposed degradation pathways of UB (**C**).
